# Current Understanding of Methamphetamine-Associated Metabolic Changes Revealed by the Metabolomics Approach

**DOI:** 10.3390/metabo9100195

**Published:** 2019-09-20

**Authors:** Minjeong Kim, Won-Jun Jang, Rupa Shakya, Boyeon Choi, Chul-Ho Jeong, Sooyeun Lee

**Affiliations:** College of Pharmacy, Keimyung University, 1095 Dalgubeoldaero, Dalseo-gu, Daegu 42601, Korea; alswjd91vov@naver.com (M.K.); mrdoin76@gmail.com (W.-J.J.); rupashakya48@yahoo.com (R.S.); qhdus1210@naver.com (B.C.); chjeong75@kmu.ac.kr (C.-H.J.)

**Keywords:** metabolomics, methamphetamine, drug addiction, brain, biological samples, metabolism

## Abstract

Metabolomics is a powerful tool used in the description of metabolic system perturbations caused by diseases or abnormal conditions, and it usually involves qualitative and/or quantitative metabolome determination, accompanied by bioinformatics assessment. Methamphetamine is a psychostimulant with serious abuse potential and due to the absence of effective pharmacotherapy and a high recurrence potential, methamphetamine addiction is a grave issue. Moreover, its addiction mechanisms remain unclear, probably due to the lack of experimental models that reflect personal genetic variances and environmental factors determining drug addiction occurrence. The metabolic approach is only recently being used to study the metabolic effects induced by a variety of methamphetamine exposure statuses, in order to investigate metabolic disturbances related to the adverse effects and discover potential methamphetamine addiction biomarkers. To provide a critical overview of methamphetamine-associated metabolic changes revealed in recent years using the metabolomics approach, we discussed methamphetamine toxicity, applications of metabolomics in drug abuse and addiction studies, biological samples used in metabolomics, and previous studies on metabolic alterations in a variety of biological samples—including the brain, hair, serum, plasma, and urine—following methamphetamine exposure in animal studies. Metabolic alterations observed in animal brain and other biological samples after methamphetamine exposure were associated with neuronal and energy metabolism disruptions. This review highlights the significance of further metabolomics studies in the area of methamphetamine addiction research. These findings will contribute to a better understanding of metabolic changes induced by methamphetamine addiction progress and to the design of further studies targeting the discovery of methamphetamine addiction biomarkers and therapeutic targets.

## 1. Introduction

Metabolomics is a powerful tool used for describing metabolic system perturbations caused by diseases or abnormal conditions. It involves qualitative and/or quantitative metabolome determination, accompanied by bioinformatics assessment [1,2]. Particularly, in recent years, the discovery of novel biomarkers using metabolomics has received a lot of attention, based on the understanding that impaired biochemical pathways related to pathophysiological conditions affect the regulation of metabolomes, which are the final products in the omics cascade [3,4,5,6,7,8]. Additionally, metabolomics has attracted increasing interest from toxicology fields because it is more straightforward in the investigation of the toxic effects and mechanisms of drugs or toxicants, compared with conventional toxicological assays [9].

Metabolomics constitutes pre- and post-analytical processes, as well as metabolic profiling, including sample preparation and instrumental analysis, using high- (HR) or low-resolution (LR) mass spectrometry (MS) and/or nuclear magnetic resonance spectroscopy (NMR). The pre-analytical process involves experimental design such as hypothesis establishment and the design of control and experimental groups in cells, animals, or humans. The post-analytical process includes data processing and metabolite/ion feature identification, followed by differential analysis (e.g., principal component analysis and partial least squares-discriminant analysis), and significance analysis (e.g., analysis of variance followed by post-hoc analysis). Furthermore, bioinformatics analyses—such as metabolite set analysis, pathway-based analysis, biological network analysis, and correlation analysis—can be performed to arrive biologically relevant conclusions [10,11,12].

**Figure 1:**
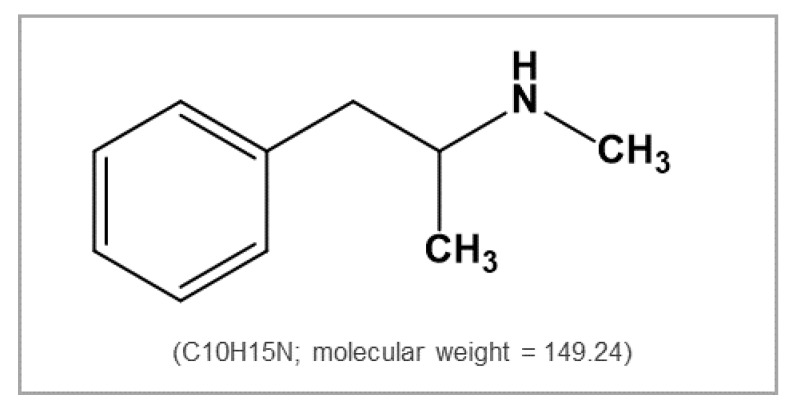
Chemical structure of methamphetamine.

**Figure 2:**
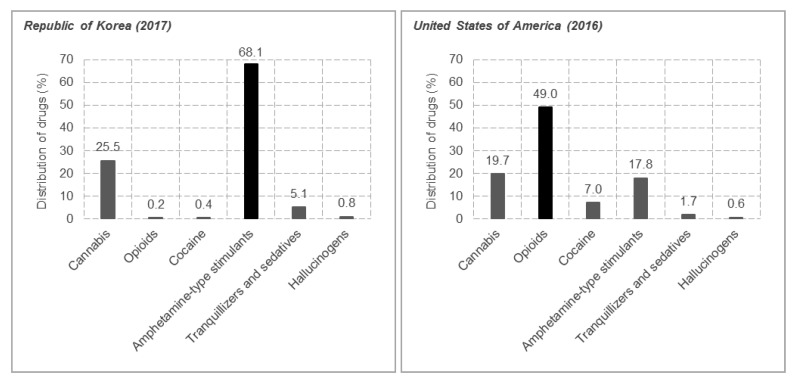
Primary drugs of abuse among persons treated for drug problems in the Republic of Korea (2017) and the United States of America (2016).

**Figure 3:**
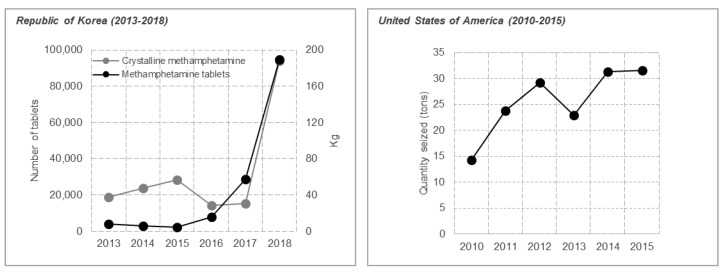
Methamphetamine-induced seizures in the Republic of Korea (2013–2018) and the United States of America (2010–2015).

Methamphetamine (Figure 1), a psychostimulant with serious abuse potential has become a critical social problem because of its increasing use since the 1990s. It is reported to be the second most abused substance following cannabis and it is estimated that 0.3–1.3% of the world’s population abuse methamphetamine [13,14]. In 2017, in the Republic of Korea, amphetamine-type stimulants (ATS), mostly methamphetamine, were the most abused drugs among persons treated for drug-related problems (68.1%), followed by cannabis (25.5%) [15]. In the United States of America, ATS was one of major problematic drugs as well [16] (Figure 2). Interestingly, methamphetamine-induced seizures increased from 30.5 to 187.9 kg and from 28,700 to 94,867 tablets between 2017 and 2018 for crystalline and tablet methamphetamine, respectively [17]. The quantities of methamphetamine seized has also increased in recent years in the United States of America [16] (Figure 3). Methamphetamine can easily pass through the blood–brain barrier, and significantly affect neurochemical systems via multiple neurotransmitter receptors and transporters [13,18]. Previous studies in rat brain have reported that the acute effects of methamphetamine are mainly due to two main molecular substrates; vesicular monoamine transporter-2 (VMAT-2) and dopamine transporter (DAT), which control dopamine release [18,19]. In addition, methamphetamine affects serotonergic, noradrenergic, and glutamatergic systems through interactions with monoamine (dopamine, serotonin, and norepinephrine) transporters and N-methyl-D-aspartate (NMDA) receptors. The release of monoamines and reduction of their reuptake provoke significant increases in their levels in extracellular spaces and excessive stimulation of the sympathetic system [13,18]. However, after repeated administration of high doses to rodents and nonhuman primates, striatal concentration of dopamine and its metabolites has been reported to decrease [18]. The first major clinical evaluation of the effects of long-term methamphetamine use demonstrated that the mean striatal (nucleus accumbens (NAc), caudate, and putamen) dopamine levels in the brain of 12 long-term methamphetamine users reduced by 39–55% [20]. Clinically, methamphetamine addiction is a grave issue due to the absence of effective pharmacotherapy and its high recurrence potential [21]. Moreover, its addiction mechanisms remain unclear, probably due to the lack of experimental models that reflect personal genetic variances and environmental factors determining drug addiction occurrence.

In the present review, the latest findings on methamphetamine exposure-related metabolic alterations are summarized. For this purpose, methamphetamine toxicity, applications of metabolomics in drug abuse and addiction studies, biological samples used in metabolomics, and previous animal studies on metabolic alterations in various biological samples such as the brain, hair, plasma, serum, and urine, following methamphetamine exposure, were reviewed and discussed. This highlighted the significance of metabolomics studies in methamphetamine addiction research.

## 2. Application of Metabolomics in Drug Abuse and Addiction Studies

Drug addiction, a chronic disease characterized by compulsive drug seeking, is associated with an increased risk of cancer, psychological complication, heart, liver, and lung disease, as well as infection [22,23]. Even though chemicals of abuse are structurally diverse and exert a variety of behavioral effects on the users, the deficit of the reward pathway is suggested as a drug addiction mechanism. When the reward pathway is activated by a stimulus, dopamine is produced in nerve cell bodies in the ventral tegmental area (VTA), and then released into the NAc, prefrontal cortex (PFC), and striatum [24,25,26,27,28]. 

Based on the theory proposed from the development of methadone maintenance programs, addiction could be considered a metabolic disease, given that it is initiated by a disruption in metabolism and causes persistent neurochemical disturbances leading to addiction [29]. The potential of metabolomics in the investigation of the toxicities of drugs of abuse and the development of predictive neuropsychiatric disorder biomarkers have recently been highlighted [2,30]. Several metabolomics studies recently conducted to investigate the effects of addictive drug use revealed that drug exposure causes metabolism imbalance. A previous study reported that energy metabolism-related metabolic profiles were changed in rat plasma and/or urine in morphine, methamphetamine, and cocaine-induced conditioned place preference (CPP) models, compared with their controls [31]. Another previous study demonstrated that after cocaine treatment (30 mg/kg, intraperitoneal (i.p.)), the concentrations of serotonin, norepinephrine, glucose, dopamine, 3,4-dihydroxyphenyl acetic acid (DOPAC), and 5-hydroxyindoleacetic acid (5-HIAA) were significantly altered in brain regions—including the thalamus, striatum, and frontal cortex—and anatomically different. This study also suggested that cocaine has an effect on the glycolysis metabolome in the thalamus [32]. A metabolomics study of the brain regions of alcohol dependent rats, established using chronic intermittent alcohol vapor exposure, proposed dopamine and met-enkephalin concentrations as possible recent drinking history indicators [33].

## 3. Biological Samples Used in Metabolomics

Metabolomics studies have been conducted using a variety of biological samples, including cells, plasma/serum, urine, and other tissues (e.g., brain), with plasma/serum and urine being the most commonly used [34]. Plasma, obtained from whole blood with anticoagulants, contains approximately 90% water, 2% inorganic and organic ions, and 8% proteins (mostly albumin), including enzymes. Serum is obtained from whole blood after coagulation, via centrifugation [35]. Plasma/serum is the preferred biological specimen for drug and metabolite analysis because the concentration of an analyte in plasma/serum is significantly associated with its pharmacological or toxicological effect. Plasma/serum has been increasingly used in biomarker discovery for diseases, probably because it contains metabolomes which reflect an organism’s current state [36,37].

Due to easy and noninvasive sampling and the presence of high quantities of metabolites, urine is considered ideal for biomarker monitoring in clinical analysis. It contains 98% water and the major solutes are anionic (carboxylic acids) or amphoteric (amino acids) in nature. In addition, it contains varying concentrations of thousands of different solutes, including endogenous metabolomes [35]. Thus, urine metabolomics has been applied in the investigation of metabolic alterations in response to a specific disease or therapeutic intervention [38,39]. However, urine volume variation, which depends on an individual’s hydration status, is an analytical challenge in the interpretation of the quantitative results of metabolomes. Urine creatinine, specific gravity, and osmolality have been used to verify the authenticity of urine samples; for example, to detect intentional dilution of urine with other fluids, in forensic urine drug analysis. The determination of creatinine-adjusted xenobiotic or metabolome concentrations in urine are required to improve the quality of the quantitative results because this correction compensates for individual variations in physiological urine dilution [39,40,41].

Hair is mainly used as an alternative specimen to prove chronic substance use. The substances and metabolites in capillaries connected to hair roots bind to melanin or sulfhydryl-containing amino acids and incorporate into hair as it grows at approximately 1 cm per month. The detection window in hair is much longer than in other biological samples and the segmental analysis of hair based on its growth rate can track substance use history over time. Additionally, hair has many advantages as a bioanalytical sample, including easy sample collection, convenient transport, and storage, and easy repetition of sampling, if necessary. Human hair is composed of fibrous proteins (mostly α-keratins, 85–93%), melanin pigments, water (typically 3–5% and up to 15% by mass), lipids (1–9%), and mineral compounds (0.25–0.95%) [42,43,44,45]. Given that it contains functional metabolomes such as amino acids and lipids, it was recently introduced as a specimen with high potential in metabolomics studies, especially for chronic disease biomarker discovery, including drug addiction and abnormal conditions [11,34,46].

Target tissue-based metabolomics have been performed in cancer tissues and other tissues such as the liver and brain, extracted from experimental animals and humans with liver metabolism disorders or brain injuries and diseases [47,48,49,50]. In spite of the limitations of tissue sample accessibility and the complexity of sample preparation, target tissue-based metabolomics still represents the most direct way to gain insights into the metabolomic changes in the cells of the target tissue [47,50]. In neurodegenerative disease and mental disorder studies, the use of brain tissue is rapidly increasing [50]. The brain consists of the cerebrum, cerebellum, and the brainstem, which have functionally different subparts. Given that the addiction-related reward pathway—whereby dopamine is released from the VTA to the NAc, PFC, and striatum in the cerebrum—is well-known [24,25,26,27,28], metabolomics in brain tissues, including these parts, could reveal drug addiction-related metabolic alterations. Brain metabolomics studies have often been conducted using NMR, which enables the non-destructive analysis of the intact brain tissue’s chemical composition, with minimal sample preparation. In human studies, the post-mortem human brain is used; thus, different post-mortem intervals affect the metabolic variation, which is the major issue in analytical result interpretation. In the analysis of brain tissues collected from experimental animals, there is still a debate regarding the use of perfusion protocols and the use of anesthesia [10,51]. Brain microdialysis has been proposed as an alternative method for monitoring brain metabolism in living animals [52].

## 4. Metabolic Alterations in Brain Following Methamphetamine Exposure in Animal Studies

Table 1 summarizes the metabolomics studies performed to investigate metabolic changes following methamphetamine exposure in the brain of mice or rats [53,54,55,56]. In these studies, locomotor activity was measured [53,54,55] after multiple methamphetamine administrations (i.p. or subcutaneous (s.c.)), and then whole brain tissue, subparts of the brain tissue, or microdialysate from the subparts were collected and analyzed [53,54,55,56]. Particularly, untargeted metabolomics in brain tissues from methamphetamine-affected animals uncovered methamphetamine-related neurochemical signatures [53,54,55]. As revealed by the untargeted metabolomics study of Adkins et al., the concentrations of betaine, glutarylcarnitine, pantothenate, and ribulose in the whole brain of methamphetamine-sensitized rats increased, while those of isovalerylcarnitine, 4-glutarylcarnitine, n-acetylglutamate, myo-inositol, and homocarnosine decreased, compared with the control group. Additionally, the associations of homocarnosine, a dipeptide composed of histidine and GABA, with total distance sensitization, 4-guanidinobutanoate, a GABA metabolite, and pantothenate with stereotypy sensitization, and myo-inositol with margin time sensitization were reported [53]. Bu et al. performed untargeted metabolic profiling in the hippocampus, NAc, and PFC of methamphetamine-sensitized rats using ¹H NMR spectroscopy. Repeated methamphetamine exposure significantly decreased succinate, n-acetylaspartate, α-ketoglutarate, citrate, methionine, glutamine, glutathione, glutamate, and γ-aminobutyric acid levels in all the hippocampus, NAc, and PFC tissues by repeated methamphetamine exposure, compared with the control group. Additionally, there was a change in the hippocampus, NAc, and/or PFC levels of taurine, phosphocholine, serotonin, acetylcysteine, homocysteic acid, myo-inositol, succinic acid semialdehyde, and dopamine. The alteration of these metabolites implied methamphetamine-induced neurotransmitter disturbance, including abnormality of glutamine and glutamate, oxidative stress and membrane disruption, and glial activation dysfunction [54]. In another previous untargeted metabolomics study of mouse whole brain, the concentration of 3-dehydrocarnitine, tryptophan, tyrosine, lactate, 2-hydroxyglutarate, fumarate, malate, and succinate were significantly increased, while those of fructose and serotonin were significantly decreased by a single dose methamphetamine administration (3 mg/kg, i.p. once per day). The concentrations of ergothioneine and phosphocholine significantly increased with repeated methamphetamine exposure for 5 days. The upregulation of fumarate, malate, and succinate is associated with altered energy metabolism, and that of 2-hydroxyglutarate supports the disruption of mitochondrial activity. Furthermore, methamphetamine-induced neuronal damage was demonstrated by the alteration of phophocholine and N-acetyl-aspartate levels [55]. In a study by Bustamante et al., the increased extracellular level of dynorphin B, an endogenous opioid peptide, probably as a secondary effect of an increased dopamine release, and the decreased levels of dopamine metabolites, DOPAC, and homovanillic acid (HVA), as well as the serotonin metabolite. 5-HIAA, was reported in in vivo microdialysates collected from substantia nigra and neostriatum [56]. However, consistent results were not indicated, distinct alteration of common metabolites were not stated, and results on the changes in the levels of some metabolites such as myo-inositol, succinate, and dopamine, were conflicting. These variations might result from differences in sampling time point, dose, and target region, as pointed out by Zaitsu et al. [57]. Regardless of these limitations, brain metabolomics studies on methamphetamine exposure offer insights on metabolic effects on brain energy metabolism and neurotransmission. Further in-depth studies on methamphetamine-induced metabolic effects, based on a variety of conditions in animal studies, are necessary.

## 5. Metabolic Alterations in Other Biological Samples (Hair, Plasma, Serum, and Urine) Following Methamphetamine Exposure in Animal Studies

Previous metabolic investigations in other biological samples (hair, plasma, serum, and urine) following methamphetamine exposure in animal studies, are summarized in Table 2 [9,11,31,58]. Animal models of self-administration and conditioned place preference (CPP) were employed in two previous studies [11,31]. In the other previous studies, repeated i.p. methamphetamine injections, followed by sampling at different time points was performed [9,58]. Self-administration is considered to be a valid and standard tool in drug addiction studies. It is determined by measuring drug seeking and drug taking behavior, using a drug-connected lever-pressing in an operant conditioning chamber. The self-administration process offers a direct relation with addictive behavior, happening in the natural environment, with features of human drug abuse modeled by the self-administration process in animals. Moreover, the self-administration process generates stable individual behaviors, enabling reliable interpretation of data generated by within-subject designs [59,60]. The CPP method, which reflects the rewarding effect of drugs of addiction by measuring the length of time an animal spends in a place that has been associated with a drug, is also widely used. However, the interpretation of its results is sometimes controversial because the multiple learning processes involved, including incentive-driven behavior related to secondary reinforcers, operant conditioning of behavior prevailing at the conditioning site, and conditioned treatment effects may be oversimplified only to drug conditioning [61,62]. Both self-administration and the CPP method have predictive validity in evaluating the abuse liability of novel compounds, and they are often used in behavioral and mechanistic studies related to drugs of abuse. However, a few metabolic changes have been reported after self-administration or CPP of addictive drugs.

Choi et al. conducted metabolic characterizations in urine and hair obtained from a rat model of methamphetamine self-administration, using untargeted liquid chromatography-HR-MS. Interestingly, more significant changes in functional metabolites were observed in hair, compared with urine. Acetylcarnitine and palmitoyl-(l)-carnitine were upregulated, while deoxycorticosterone, oleamide, and stearamide were downregulated, implying metabolic perturbations in the central nervous system and energy production. Based on these findings, animal hair was proposed as a more reliable diagnostic specimen for drug addiction studies [11]. In another study, a methamphetamine-induced CPP model was established using rats and metabolic profiling was performed on urine and plasma. The concentrations of n-propylamine in plasma significantly increased, while that of lauric acid significantly decreased. Additionally, the urine levels of lactose, spermidine, and stearic acid significantly increased [31]. Zheng et al. investigated the changes in the levels of amino acids in rat serum following the administration of a single dose (10 mg/kg, i.p.) or escalating doses (10–30 mg/kg, i.p.) of methamphetamine for 5 days consecutively, followed by 2 days abstinence, using gas chromatography (GC)-MS, after chemical derivatization. The levels of most amino acids, including alanine, glycine, and three branched chain amino acids (valine, leucine, and isoleucine), decreased significantly after the single and/or escalating administration, while those of glutamate and lysine increased. After the 2 days of abstinence, most of the amino acid levels were restored. The depletion of the three branched-chain amino acids indicated that they had been consumed in large quantities for energy supply. The authors also suggested that alterations in the levels of other amino acids were related to nervous system activation. Additionally, alterations of the metabolites in the tricarboxylic acid (TCA) cycle and lipid metabolism have been demonstrated [58]. Furthermore, the impairment of energy metabolism by methamphetamine exposure was demonstrated in a study conducted by Shima et al., in which rats were treated with methamphetamine at 10 mg/kg/h (i.p.) for 4 h. Thereafter, plasma and urine collected at 24 and 96 h (for plasma) and between 0–24 and 72–96 h (for urine) after the last administration were analyzed using GC-HR-MS and capillary electrophoresis-MS/MS. Methamphetamine administration increased plasma glucose level and decreased urinary TCA cycle intermediates and fructose 1,6-bisphosphate levels, indicating metabolic perturbation in glycolysis, the TCA cycle, and fatty acid metabolism. The urine sample collected between 72–96 h, after the last drug administration, revealed that the levels of all the urinary metabolites affected by methamphetamine exposure recovered to the control levels [9]. 

The results of non-separative PCA clustering in plasma and urine in methamphetamine-induced CPP have been reported [31]. However, clear differentiation from the control was shown in both rat plasma and urine by acute methamphetamine (i.p., 10 mg/kg/h, 4 h) intoxication [9] and increasing methamphetamine doses for 5 consecutive days (10, 12.5, 15, 20, and 30 mg/kg for each day) [58]. These authors proposed a possible mechanistic difference between methamphetamine addiction and acute intoxication and physiological adaption during chronic methamphetamine use [31]. Conversely, the PCA revealed that clear clustering was shown by hair samples collected before and after methamphetamine self-administration [11].

## 6. Perturbed Metabolic Pathways Associated with Methamphetamine Exposure

**Figure 4:**
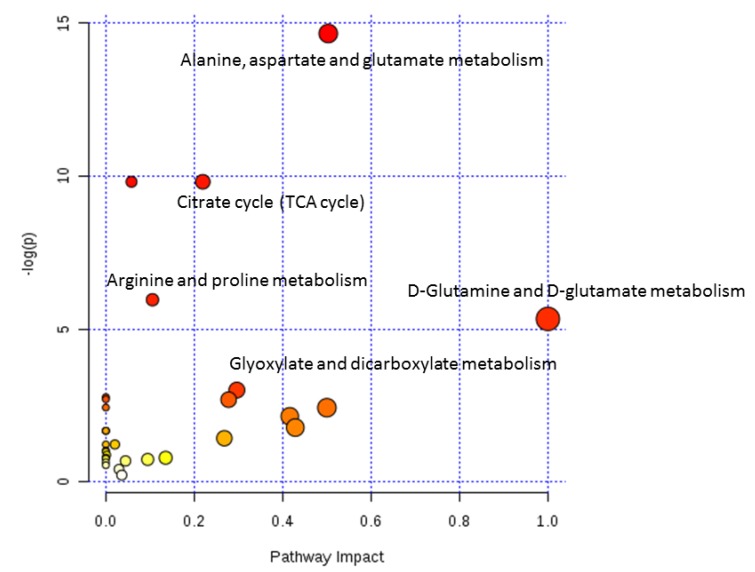
Result of metabolic pathway analysis with MetaboAnalyst. The size and color of each circle represent pathway impact value and *p*-value, respectively.

The metabolites with significantly changed concentrations following methamphetamine exposure, shown in Table 1 and Table 2, were inconsistent, depending on the doses and durations of methamphetamine administration and targeted animal model samples. To understand the alteration of the metabolites found in previous studies, we performed a metabolic pathway analysis with a few selected studies [59,60,61]. These selected studies aimed to investigate brain metabolic changes related to behavioral effects induced by medium- or low-dose methamphetamine in mice or rats. The metabolic pathway analysis was performed using the online MetaboAnalyst 4.0 software (http://www.metaboanalyst.ca/MetaboAnalyst/faces/home.xhtml) and the results are shown in Figure 4 and summarized in Table 3. Methamphetamine exposure disturbed the citrate cycle (TCA cycle), as well as the metabolism of alanine, aspartate and glutamate, arginine and proline, D-glutamine and D-glutamate, and glyoxylate and dicarboxylate. The metabolism of alanine, aspartate and glutamate was most significantly affected (*p* = 4.2753 × 10^−7^). Aspartate and glutamate are known as major excitatory neurotransmitters. The biosynthesis of these amino acids is also associated with the intermediates of the citrate cycle and the metabolic pathway analysis results revealed that this cycle was significantly perturbed.

## 7. Future Directions

Despite the recent growth of the application of metabolomics to understanding the effect of methamphetamine on the metabolic system, there are numerous opportunities for enhancing and expanding the impact of metabolomics in methamphetamine addiction research. Since the symptoms and severities of methamphetamine toxicity—including drug dependence and withdrawal—are diverse, further studies using well-designed animal models that reflect specific conditions or phenotypes of methamphetamine toxicity are necessary. Currently, human metabolomics data in this area are limited, compared with data for other metabolic diseases. Methamphetamine-related potential biomarkers proposed in animal studies need to be validated in clinical settings. Furthermore, bioinformatics analyses for methamphetamine-associated metabolomics data have seldom been performed, compared with other-omics data. A variety of bioinformatics approaches—such as metabolite set analysis, pathway-based analysis, biological network analysis, and correlation analysis—could facilitate comprehensive understanding of the biological functions of metabolic alterations caused by methamphetamine exposure.

## 8. Conclusions

**Figure 5:**
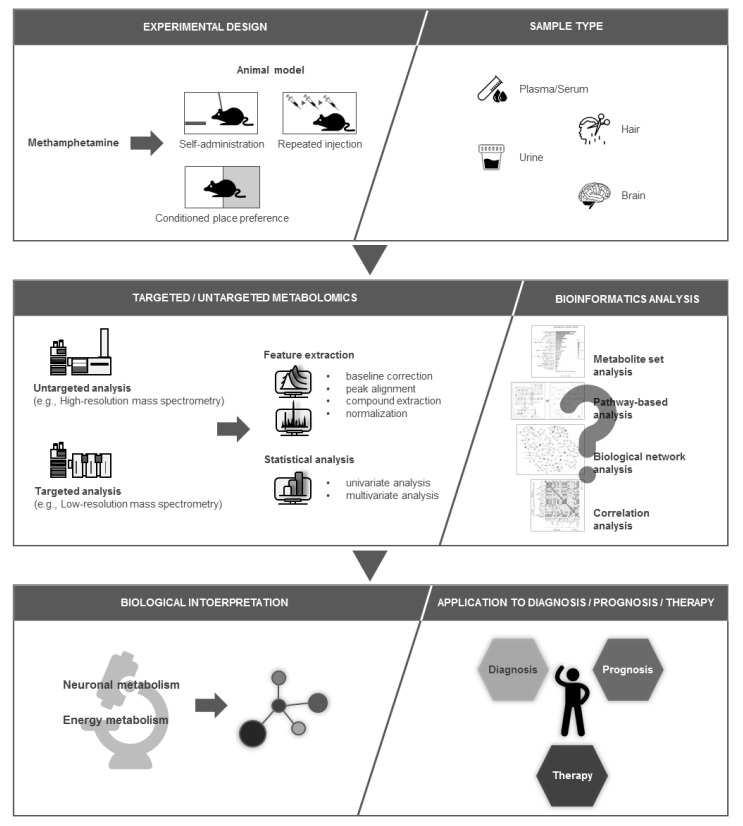
Application of metabolomics in methamphetamine addiction research.

As shown in Figure 5, metabolomics approaches have recently been employed in the investigation of metabolic perturbation, following a variety of methamphetamine exposure statuses (e.g., self-administration, CPP, and repeated administration) in not only brain tissues but also in other biological samples, such as plasma/serum, urine, and hair. The metabolic alterations observed in these recent studies were associated with neuronal and energy metabolism disruptions. This review highlights the significance of further metabolomics studies in methamphetamine addiction research. These findings will contribute to a better understanding of the metabolic changes induced by progress in methamphetamine addiction and to the design of further studies targeting the discovery of methamphetamine addiction biomarkers and therapeutic targets.

## Figures and Tables

**Table 1:** Summary of metabolic changes following methamphetamine exposure in animal brain

Reference No.	No.	Animal	Sample	Analytical Platform (Untargeted Or Targeted)	Experimental Condition (Administration Dose, Route, Times, Sampling Time, etc.)	Metabolic Changes	Metabolic Effects
[53]	1	Mouse	Whole brain	LC-(HR)MS and GC-MS (Untargeted)	3 mg/kg, i.p., once a day for 5 days Sampling on Day 5 after 1-h locomotor activity measurement	Isovalerylcarnitine (↓), myo-inositol (↓), betaine (↑), glutarylcarnitine (↑), ribulose (↑), pantothenate (↑), n-acetylglutamate (↓), homocarnosine (↓), and 4-guanidinobutanoate (↓)	Neurochemical alteration by methamphetamine-induced psychomotor sensitization
[54]	2	Rat	Hippocampus, NAc, and PFC	^1^H NMR (Untargeted)	2.5 mg/kg, s.c., 2 times/day (10-h intervals) for 7 days Locomotor activity measurement on Days 0, 1, 3, and 5 after the second injection Sampling on Day 7	Hippocampus, NAc and PFC: Succinate (↓), n-acetylaspartate (↓), α-ketoglutarate (↓), citrate (↓) methionine (↓), glutamine (↓), glutathione (↓), glutamate (↓) and γ-aminobutyric acid (↓) NAc and PFC: Taurine (↓), phosphocholine (↑) and serotonin (↓) Hippocampus and NAc: Acetylcysteine (↓) and homocysteic acid (↑) Hippocampus and PFC: Myo-inositol (↑) Hippocampus: Succinic acid semialdehyde (↑) NAc: Dopamine (↓)	Disturbance in neurotransmitters, oxidative stress, membrane disruption, and glial activation
[55]	3	Mouse	Whole brain	LC-(HR) and GC-MS (Untargeted)	3 mg/kg, i.p., once a day for 1 day (D1) or for 5 days (D5) Sampling after 1-h locomotor activity measurement after the last drug administration	D1: 3-dehydrocarnitine (↑), tryptophan (↑), serotonin (↓), tyrosine (↑), fructose (↓), lactate (↑), 2-hydroxyglutarate (↑), fumarate (↑), malate (↑) and succinate (↑) D5: Ergothioneine (↑), and phosphocholine (↑)	Increased energy metabolism, disrupted mitochondrial activity, and neuronal damage
[56]	4	Rat	Microdialysate from substantia nigra and neostriatum	LC-ECD for monoamines, LC-FLD for amino acids, and dynorphine B radioimmunoassay (targeted)	15 mg/kg, s.c., three times at 9, 15, and 21 h Sampling after 12 h from the last administration every 40 min during 4 h, microdialysis	Dopamine (↑), 3,4-dihydroxyphenylacetic acid (↓), homovanillic acid (↓), 5-hydroxyindoleacetic acid (↓), and dynorphin B (↑)	Impairment of monoamine neurotransmission and changes in amino acid homeostasis

LC-MS, liquid chromatography-mass spectrometry; HR, high resolution; GC-MS, gas chromatography mass spectrometry; LC-ECD, liquid chromatography-photodiode array detector; i.p., intraperitoneal injection; s.c., subcutaneous injection; NAc, nucleus accumbens; PFC, prefrontal cortex; ↓, significantly decreased vs. vehicle group; ↑, ↓, significantly decreased vs. vehicle group.

**Table 2:** Summary of significantly metabolites changes following methamphetamine exposure in other samples

Reference No.	No.	Animal	Sample	Analytical platform (Untargeted Or Targeted)	Experimental Condition (Administration Dose, Route, Times, Sampling Time, etc.)	Metabolic Changes	Metabolic Effects
[11]	1	Rat	Hair	LC-(HR)MS (Untargeted)	Self-administration (i.v., 0.05 mg/kg/injection, 2 h/day, 16 days) Sampling at each of before and after self-administration	(L)-norvaline/betaine/5-aminopentanoate/(L)-valine (↓), acetylcarnitine (↑), 5-methylcytidine (↑), 1-methyladenosine (↑), lumichrome (↓), Cys Arg Met (↓), palmityl-L-carnitine (↑), deoxycorticosterone (↓), oleamide (↓), stearamide (↓), and hippurate (↓)	Metabolic perturbation in the central nervous system and energy production
[31]	2	Rat	Plasma	GC-MS (Untargeted)	Conditioned place preference (2 mg/kg, i.p., once a day, 2 days for pre-priming, 10 days for conditional training, and 2 days for post-priming) Sampling after post-priming	N-propylamine (↑) and lauric acid (↓)	No changes in many metabolites probably due to adaptations to chronic methamphetamine administration
			Urine			Lactose (↑), spermidine (↑) and stearic acid (↑)	
[58]	3	Rat	Serum	GC-MS (Untargeted)	10, 12.5, 15, 20, and 30 mg/kg (escalating dose for 5 days), i.p. Sampling after 1 h on Day 1 (D1) and 5 (D5) and after withdrawal of 2 days (W)	D1: Glycine (↓), valine (↓), isoleucine (↓), leucine (↓), α-ketoglutarate (↓), succinate (↓), citrate (↓), pyruvate (↓), myo-inositol-1-phosphate (↓), indoleacetate (↓) and ^1^H-indole-3-propanoic acid (↑) D5: Monopalmitin (↓), 3-hydroxybutyrate (↑) and stearic acid (↓) *D1: Alanine (↓), asparagine (↓), citrulline (↓), glutamate (↑), glycine (↓), proline (↓), ornithine (↓), serine (↓), threonine (↓), valine (↓), leucine (↓), isoleucine (↓), hydroxyproline (↓), taurine (↓), methionine (↓), lysine (↑), ketoleucine (↓), monopalmitin (↓), cis-9-hexadecenoic acid (↑), 3-hydroxybutyrate (↑), glycerol (↑), glycerol-3-phosphate (↓), aminomalonic acid (↓), α-ketoglutarate (↓), citrate (↓), pyruvate (↓), succinate (↓), galactonolactone (↑), creatinine (↓), indoleacetate (↓), myo-inositol (↓), myo-inositol-1-phosphate (↓), and lactate (↓) *D5: Alanine (↓), citrulline (↓), proline (↓), ornithine (↓), threonine (↓), isoleucine (↓), hydroxyproline (↓), methionine (↓), lysine (↑), monopalmitin (↓), palmitic acid (↓), heptadecanoic acid (↓), cis-9-Hexadecenoic acid (↓), 3-hydroxybutyrate (↑), stearic acid (↓), glycerol-3-phosphate (↓), α-aminoisobutyrate (↓), α-ketoglutarate (↓), citrate (↓), pyruvate (↓), galactonolactone (↓), creatinine (↓), and myo-inositol-1-phosphate (↓) *W: Isoleucine (↓), lysine (↑), palmitic acid (↓), cis-9-Hexadecenoic acid (↓), α-aminoisobutyrate (↓), α-ketoglutarate (↓), citrate (↓), succinate (↓), galactonolactone (↑), and creatinine (↓)	Elevated energy metabolism, TCA cycle and lipid metabolism, and activation of nervous system
			Urine			D5: 3-Hydroxybutyrate (↑) and glycerol (↑) *D5: Serine (↑), glutamate (↑), alanine (↑), 3-hydroxybutyrate (↑), hippurate (↓), lactate (↑), galactonate (↑), pyruvate (↑), fumarate (↑), succinate (↑), myo-inositol (↑), and 5-hydroxyindoleacetic acid (↓) *W: Hippurate (↓) and lactate (↑)	
[9]	4	Rat	Plasma	GC-TOFMS, CE-MS/MS	10 mg/kg, i.p., once per hour, 4 times Sampling after 24 h (A) and 96 h (B)	A: Glucose (↑) and 3-hydroxybutyrate (↓) B: all of the metabolites in A recovered to control levels.	Impaired energy metabolism (glycolysis, TCA cycle, and fatty acid metabolism)
			Urine		Same experimental condition as plasma collection except for sampling for 0-24 h (A) and 72-96 h (B) after the last administration	A: Citrate/isocitrate (↓), saccharic acid (↑), uracil (↑), adipic acid (↓), aconitate (↓), fumarate (↓), malate (↓), succinate (↓), 5-oxoproline (↑), α-ketoglutarate (↓), oxaloacetate/pyruvate (↓), and 3-hydroxybutyrate (↓) B: all of the metabolites in A recovered to control levels.	

LC-MS, liquid chromatography-mass spectrometry; HR, high resolution; GC-MS, gas chromatography mass spectrometry; GC-TOFMS, gas chromatography-time-of-flight mass spectrometry; CE-MS/MS, capillary electrophoresis-tandem mass spectrometry; i.p., intraperitoneal injection; ↓, significantly decreased vs. vehicle group, except for *; ↑, ↓, significantly decreased vs. vehicle group except for *; *vs. pre-administration group.

**Table 3:** Altered metabolism pathways following methamphetamine exposure

Metabolic Pathway	Total	*p*	Impact	Hits	Metabolites
Alanine, aspartate, and glutamate metabolism	24	4.2753 × 10^−7^	0.50315	7	N-Acetylaspartate, glutamate, α-ketoglutarate, γ-aminobutyric acid, fumarate, succinic acid semialdehyde, succinate
Citrate cycle (TCA cycle)	20	5.4591 × 10^−5^	0.21929	5	Succinate, fumarate, malate, citrate, α-ketoglutarate
Arginine and proline metabolism	44	2.5868 × 10^−3^	0.10545	5	Fumarate, glutamate, γ-aminobutyric acid, 4-guanidinobutanoate, N-acetylglutamate
D-Glutamine and D-glutamate metabolism	5	4.8373 × 10^−3^	1.0	2	Glutamate, α-ketoglutarate
Glyoxylate and dicarboxylate metabolism	16	4.9634 × 10^−2^	0.2963	2	Citrate, malate

Total, number of metabolites in the reference pathway in Kyoto Encyclopedia of Genes and Genomes (KEGG); hits, number of metabolites reported in [53,54,55].
